# Sex-specific DNA methylation and transcription of *zbtb38* and effects of gene–environment interactions on its natural antisense transcript in zebrafish

**DOI:** 10.1080/15592294.2023.2260963

**Published:** 2023-10-02

**Authors:** Fabien Pierron, Flore Daramy, Débora Heroin, Guillemine Daffe, Aurélien Barré, Olivier Bouchez, Macha Nikolski

**Affiliations:** a Univ Bordeaux, CNRS, Bordeaux INP, Pessac, France; b Univ Bordeaux, CNRS, La Rochelle Univ, INRAE, Pessac, France; c Univ Bordeaux, Bordeaux Bioinformatics Center, Bordeaux, France; d INRAE, US 1426, GeT-PlaGe, Genotoul, Castanet-Tolosan, France; e Univ Bordeaux, CNRS, IBGC, Bordeaux, France

**Keywords:** Sex determination, epigenetics, offspring sex ratio, environment, transgenerational, pollutant

## Abstract

There is increasing evidence for the involvement of epigenetics in sex determination, maintenance, and plasticity, from plants to humans. In our previous work, we reported a transgenerational feminization of a zebrafish population for which the first generation was exposed to cadmium, a metal with endocrine disrupting effects. In this study, starting from the previously performed whole methylome analysis, we focused on the *zbtb38* gene and hypothesized that it could be involved in sex differentiation and Cd-induced offspring feminization. We observed sex-specific patterns of both DNA methylation and RNA transcription levels of *zbtb38*. We also discovered that the non-coding exon 3 of *zbtb38* encodes for a natural antisense transcript (NAT). The activity of this NAT was found to be influenced by both genetic and environmental factors. Furthermore, increasing transcription levels of this NAT in parental gametes was highly correlated with offspring sex ratios. Since *zbtb38* itself encodes for a transcription factor that binds methylated DNA, our results support a non-negligible role of *zbtb38* not only in orchestrating the sex-specific transcriptome (i.e., sex differentiation) but also, via its NAT, offspring sex ratios.

## Introduction

Despite the fact that sex ratio is a simple and basic demographic parameter, it provides an essential information on the future breeding potential of a population and thus, on population viability. The production of males and females in a 1:1 ratio is generally the most common evolutionary stable strategy [1]. However, in some cases, environmental factors directly regulate/affect sex ratios [1,2]. Even if skewed sex ratios can be the direct consequence of natural or anthropogenic selection [1,3], this response can also be plastic, both in species with genotypic or environmental sex determination (GSD or ESD, respectively) [4,5]. In species with ESD, sex determination is driven by environmental factors such as photoperiod, temperature, hypoxia, population density, or toxicants [4]. Likewise, in GSD species, primary genetic sex can also be challenged by environmental cues, a phenomenon that is observed in different phyla and relatively common in insects, fish, amphibians, and reptiles [4–6]. The most known example of ESD is temperature-dependent sex determination (TSD), where the thermal conditions experienced during the early stages of development of an organism influence its sex differentiation and development [7]. In groups with TSD, extreme sex-ratio biases triggered by exposure to high temperatures are considered to be an important extinction driver [8,9]. Despite the fact that ESD is frequently presented as the result of an exposure that occurs at early developmental stages (i.e., during primordial germ cell development and gonad growth), exposure of parents (i.e., when the sex is already established and gonads are fully matured) can also affect the sex ratio of their offspring [10,11], also called sex ratio at birth [12]. Understanding the drivers and underlying mechanisms that trigger variations in offspring sex ratios is a long-standing challenge with important population implications [8].

In our previous work, we reported a transgenerational feminization of a zebrafish population exposed transgenerationally to cadmium (Cd), a non-essential metal with endocrine disruptive effects [10]. Sex in zebrafish is primarily determined by genetic factors (wild zebrafish possesses a ZW/ZZ sex determination mechanism while domesticated strains rather present a polygenic sex determination system [13,14]) but environmental factors can affect the sex ratio of populations [6]. In our case, the sex ratio (% males) of the Cd population passed from 57.2 ± 6.2% at the F0 generation to 36.4 ± 2.9% at the F3 generation. Only fish from the first generation (F0) were exposed to Cd. Change in sex ratios across the successive generations was found to be associated with Cd-induced changes in the promoter methylation levels of genes involved in sex differentiation, namely *cyp19a1a* and *foxl2a*, in female gonads. *Cyp19a1a* encodes for the enzyme responsible to the conversion of androgens into oestrogens, while *foxl2a* is involved in ovarian maintenance [15,16]. Thus, our previous work, as well as others [4], highlighted a relevant role of epigenetics in sex differentiation and maintenance, notably in zebrafish [17]. In our case, we also reported a highly significant relationship between the methylation level of *foxl2a* in mother gonads and offspring sex ratios, supporting the fact that the metallic pollution experienced by mothers can affect the sex of their offspring via methylation changes. However, for both *cyp19a1a* and *foxl2a*, we also reported sex-specific patterns, i.e., methylation levels of both genes were significantly different between males and females. The mean methylation level of *cyp19a1a* in female gonads reached 40.4 ± 1.3%, while it reached 94.3 ± 0.3% in male gonads. Thus, since zebrafish embryos inherit the methylome of their parents [18,19], i.e., both a hyper- and hypo-methylated allele, understanding how the methylation level of parental genes involved in sex differentiation can influence the sex of their offspring remains challenging.

The objective of the present work was to gain in-depth insight into the involvement of DNA methylation in Cd-induced transgenerational feminization of zebrafish, as well as into the mechanisms underlying these processes. In our previous work [10], we focused our analysis on genes that are already known to be involved in sex differentiation. In the present work, we used the results of a whole, without *a priori*, methylome (MeDip-Seq) approach that was carried out on larvae and adult fish of the first two generations in order to identify regions that were differentially methylated (called DMRs) in response to Cd exposure [20,21]. Among the Cd-induced DMRs, one DMR located in the intron 2 (in2) of the *zbtb38* gene retained our attention. Despite the fact that little is known about this gene, two recent reports [22,23] in the channel catfish (*Ictalurus punctatus*) highlighted a potential role of *zbtb38* in sex determination and/or differentiation. Moreover, *zbtb38* encodes for a transcription factor that binds methylated DNA in a sequence-specific context [24], regulating the activity of its target genes according to the methylation status of their promoters. Sex determination is initiated by balancing the transcription network towards the male or female fate, by activating the male genes (e.g., *dmrt1*, *amh*) and repressing female genes (e.g., *cyp19a1a*, *foxl2*), or inversely [25]. As we previously observed sex-specific DNA methylation patterns of genes involved in sex differentiation, we thus hypothesized that *zbtb38* could play a pivotal role in orchestrating the sex-specific transcriptome. To test our hypothesis, we initially intended to analyse the methylation level of the *zbtb38* DMR (intron 2) by using a targeted bisulphite sequencing method in fish gonads of all four generations. However, we found that methylation changes were associated with genetic variations which led us to rather analyse Cd-induced genetic changes. We then assessed the methylation level of the downstream sequence (i.e., exon 3) and we found that this exon (i) presents a highly sex-specific DNA methylation pattern and (ii) encodes a non-coding antisense transcript. Finally, we analysed the transcription level of this antisense transcript in regard to offspring sex ratios.

## Methods

### General rearing and breeding program

**Figure 1.**
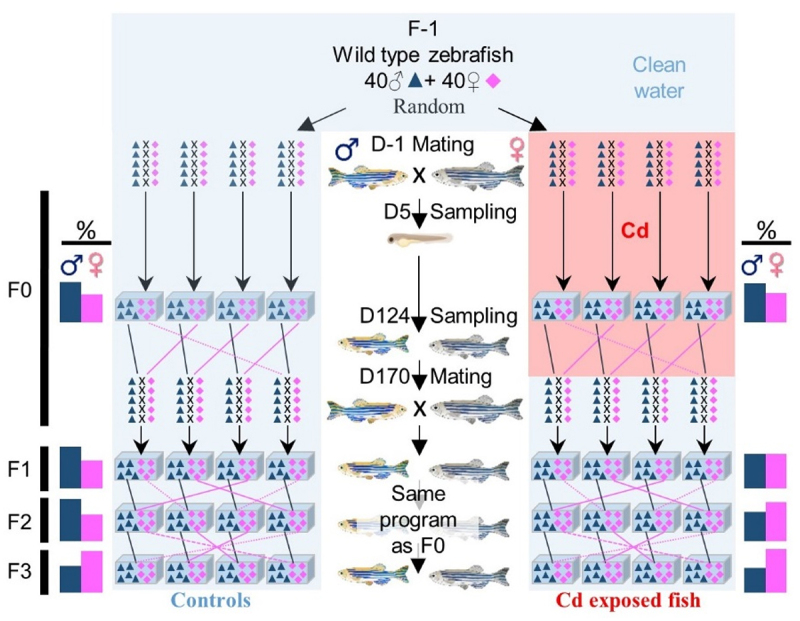
Synopsis of the experience.

The experimental set-up is presented in Figure 1, and a detailed description is available in Pierron et al. [10]. For reproduction, we used 40 small breeding tanks (BT, 2 L). Each BT was filled with one mating pair (one male and one female). At the beginning of the experiment, 40 random mating pairs were placed in tanks, out of which 20 BTs were filled with clean water (Control, C) and 20 BTs with Cd-contaminated water (1 µg.L^−1^). Their offspring (F0 generation) were, respectively, reared in clean or Cd-contaminated water throughout their life. At 14 days post-fertilization (dpf), juveniles were placed in four large aquaria (75 L) per condition permanently supplied with clean or contaminated water. Each large aquarium was filled with the larvae coming from at least three (considering that some pairs did not spawn) to maximum five different pairs. At 170 dpf, 20 pairs per condition were used to produce the next generation (F1). All mating pairs were placed in BTs filled with clean water. Thus, fish were no more exposed to Cd, and only fish of the F0 generation were directly exposed to dissolved Cd. In order to avoid inbreeding, pairs were not randomly selected: as all individuals from one clutch (i.e., from one pair) were raised in the same large aquarium, one male from one aquarium was mated with a female from another aquarium to limit inbreeding and potential genetic biases. The same protocol as previously described was used for mating for generations F1, F2, and F3. For both groups of animals (C and Cd), spawning and rearing were carried out in Cd-free water.

Only fish from the F0 generation were exposed to cadmium (Cd) throughout their life (0 to 169 dpf). At each generation and for each condition (control or Cd), we used 20 mating pairs and 4 large aquaria. Mean sex ratios (males and females %) are reported for each condition and generation. See Pierron et al., [10] for more details.

Some fish were sampled at 5 days post-fertilization (dpf, larvae) and at 124 dpf (adult mature fish). Samples (whole larvae or gonads, liver and brain from adults) were fixed in RNAlater solution before being stored at −20°C until analyses. At each generation, all remaining adults after mating (170 dpf) were sexed.

All procedures used in this experiment were approved by the Aquitaine fish-birds ethic committee (APAFIS#7535–2016111009351504).

### RNA/DNA extraction

For each generation and condition, we used six biological replicates. We used six larvae (5 dpf) from six different mating pairs and six males (124 dpf) and six females (124 dpf) from three different aquaria in order to analyse individuals from different parents. Total RNA and DNA were purified using the AllPrep DNA/RNA kit (Qiagen) according to manufacturers' guidelines.

### Identification of intergenerational Cd-induced DMRs by MeDip-Seq

In order to identify without *a priori* the regions of the genome that could be differentially methylated in response to Cd, a MeDip-Seq (methylated DNA immunoprecipitation coupled with high throughput pyrosequencing) approach was used on larvae and adult zebrafish of the F0 and F1 generation, for the total of 168 samples. Library preparation, quality checks, sequencing, and bioinformatic analyses were already described in Pierron et al. [20].

### Targeted bisulphite sequencing

Genomic sequences were obtained from the Genome Reference Consortium Zebrafish 11 (GRCz11). Specific primers used for PCR amplification and bisulphite sequencing were designed in the intron 2 (in2) and exon 3 (ex3) of the *zbtb38* gene by means of the Pyromark assay design software (Table S1).

For each sample, quantification of single cytosine percent methylation at specific CpG sites was performed by bisulphite-pyrosequencing using the PyroMark Q48 Autoprep instrument (Qiagen) as previously described in Pierron et al. (2021) [10]. In order to assess the conversion efficiency of the bisulphite treatment, the average methylation level of one CpT site was measured in another gene (*esr1*, see Pierron et al. [21]) and was <4%, indicating very good conversion efficiency. Methylation level of three different CpG sites was quantified in the ex3, and mean methylation level of these three CpG sites was used for statistical analyses. Examples of pyrograms are provided in Fig. S1. Methylation level of *cyp19a1a* in larvae was measured as previously described in Pierron et al. [10].

### Cloning and sequencing of genetic/transcript variants

Since we suspected genetic variations in the in2, we designed primers (Table S1) to amplify and sequence the in2 using native (untreated DNA) genomic DNA as a template. Specific primers were designed using the primer3plus software [26]. Amplified products (Pyromark PCR kit Qiagen) were cloned into pGEM-T Easy vector according to the manufacturer’s instructions (Promega) before to be sequenced by Sanger (GATC Biotech).

In order to identify potential transcript variants, several primers overlapping different exons were designed using the primer3plus software (Table S1). Additional variants of the *zbtb38* mRNA were identified using 3’RACE. First-strand cDNA was synthesized using the GoScript™ Reverse Transcription System (Promega) and an anchored oligo-dT (480 nM final, Table S1). A touch-down strategy was used as follows: 94°C for 15 minutes; 12 cycles of 94°C for 30 s, 64.8–60.4 for 30 s and 72°C for 3 minutes; 35 cycles of 94°C for 30 s, 60°C for 30 s and 72°C for 3 minutes and final extension at 72°C for 10 minutes using a specific forward or reverse primer with a primer complementary to the anchored oligo-dT primer (Table S1). Products were separated and visualized by gel electrophoresis. Amplified products of interest were cloned and sequenced as described above.

### Genotyping

Since we observed (see above) or suspected the presence of genetic variants in the in2, ex3 and ex4, all individuals were genotyped. For the in2 and ex4, specific primers were designed to amplify specifically the reference sequence (REF, from the GRCz11 database) or the alternative (alt) sequence. Genomic DNA from each sample was amplified with two couples of primers (REF and alt). Detection was performed either by qPCR for the in2 or PCR followed by capillary electrophoresis for the ex4 (see details in Fig. S2 and S3). We used 40 ng of DNA per sample. Thermal conditions were as follows: 95°C for 2 min; 40 cycles of 95°C for 15 s, 62°C for 2 min for the in2; or 95°C for 15 min, 40 cycles of 95°C for 1 min, 64°C for 30 s, 72°C for 1 min; 72°C for 10 min for the ex4. For the ex3, SNP detection was carried out during the run of bisulphite pyrosequencing (AQ mode, Fig. S1).

### Quantitative analysis of transcription levels

RT-qPCR analyses were carried out as previously described in Pierron *et al*. (2021) [10]. Relative quantification of gene transcription was achieved by concurrent amplification of the *eef1a1a* and *rpl13a* endogenous controls [27]. The *eef1a1a* gene was finally used as reference (see Pierron et al., 2021 [10]). Amplification efficiency for all primer sets was calculated and used to determine transcription levels of targets. Primers used are available in Table S1.

### Statistical analyses

Comparisons among fish groups were performed by two-way analysis of variance (ANOVA), after checking the assumptions of normality (Kolmogorov-Smirnov) and homoscedasticity of the error terms (Levene). If significant effects were detected, the least significant difference (LSD) test was used to determine whether means between pairs of samples were significantly different. When the assumptions were not met as deduced from the ad-hoc tests, we used box-cox data transformations or the nonparametric Kruskal−Wallis test. Using Kruskal−Wallis, the post-hoc Conover–Iman test was used. Comparisons of proportions were performed using the Monte Carlo method using 5000 permutations followed by the Marascuilo procedure to compare proportions among different conditions. Computations were performed using STATISTICA version 6.1 software (StatSoft) and XLSTAT (Addinsoft version 2020.1.1). Numerical results are reported as mean ± SE (standard error).

## Results and discussion

### Cd effect on the zbtb38 gene

#### Genome wide identification of Cd-induced differentially methylated regions

In order to identify without *a priori* the regions of the genome that could be differentially methylated in response to Cd, we first used a MeDip-Seq approach on both larvae and adult zebrafish of the F0 and F1 generation. As previously described in Pierron et al. [20,21] the two main results of this approach relied on the fact that (i) only few Cd-induced DMRs were common to the two generations and (ii) the number of Cd-induced DMRs was greater in F1 fish (i.e., not directly exposed to Cd) in comparison to F0 fish (i.e., directly exposed to dissolved Cd). Moreover, more than 12% of the Cd-induced DMRs identified in larvae of the F1 generation were found in all the three organs (liver, brain, and gonads) of F1 adult females while reaching only 2% in males. This latter result retained our attention. Firstly, we observed a feminization of the Cd population from the F1 generation. Secondly, sex differentiation must occur at an early stage of development, and the signal must be thereafter maintained throughout the development and across organs, leading to sexual dimorphism in the gonad but also in the liver or brain transcriptome [28]. Among the female developmentally conserved DMRs, one was located in the intron 2 (in2, position 41,216,101–41216400 in NC_007129.7 (chromosome 8), GRCz11) of the *zbtb38* gene. This gene encodes for a zinc finger transcriptional factor that binds methylated DNA in a sequence-specific context. Recent reports in the channel catfish (*Ictalurus punctatus*) highlighted a potential role of *zbtb38* in sex determination and/or differentiation. Channel catfish has an XY sex determination system. Yang et al. (2022) [22] reported drastic differences in DNA methylation between male and female catfish within the sex determination region (SDR) of sex chromosomes. Within this SDR, *zbtb38* was found to be differentially methylated between males and females throughout catfish development. In parallel, Pan et al. (2022) [23] using the results of sex-related quantitative trait locus (QTL) mapping and targeted next-generation sequencing identified various single nucleotide polymorphisms (SNPs) in the coding region of the *zbtb38* gene. Some of these SNPs were male-specific, allowing the development of a PCR-biased genetic sex identification method. These recent reports thus suggest an important role of *zbtb38* in sex determination and differentiation in fish. We thus decided to assess the methylation level of the in2 of the *zbtb38* gene in zebrafish from all the four generations by means of a targeted and base-resolution method, i.e., bisulphite pyrosequencing (BS-Seq).

#### Changes in DNA methylation levels within the in2 of zbtb38 were associated with genetic variations

**Figure 2.**
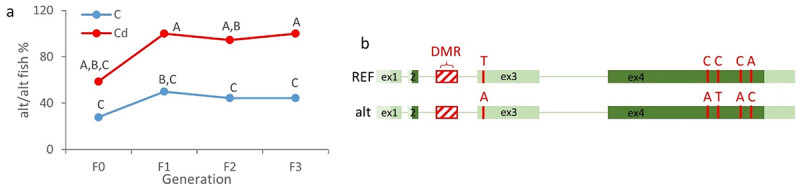
Cd exposure induced changes in allele frequencies.

Unfortunately, we failed to develop a BS-Seq method that would yield satisfactory results for the in2. While the designed sequencing primers allowed to amplify a single target, bisulphite sequencing results were of low quality, hinting at genetic variations. Thus, we then amplified the in2 using native (untreated DNA) genomic DNA as a template to analyse these genetic variations. These sequencing results allowed to identify two different sequences. The first one was 100% identical to the sequence available in the zebrafish reference genome GRCz11. This sequence will be thereafter termed as REF. The second one, called alt (alternative), showed a high homology with the REF sequence (80% identities, 3% gap) but presented comparatively a higher similarity with the sequence from the wild-derived zebrafish strain Nadia (NA, 97% identities, 0% gap, Fig. S4). We then genotyped all individuals using primers specific to the REF or alt sequences in order to test whether Cd or sex could be associated with different genotypic frequencies. As shown in Figure 2a, Cd exposure was associated with F1 generation with a significant increase in the proportion of fish presenting an alt/alt genotype. From the F1 generation, Cd fish were almost 100% homozygous alt/alt, whereas control fish were mainly heterozygous (the proportion of REF/alt control fish reaching 43.1%, REF/REF 15.3%, and alt/alt 41.7%). Moreover, no significant variation in fish genotype frequencies was observed across generations in the case of controls. Thus, our results highlight the fact that Cd exposure led to an increased frequency of the alt allele in the population. In other words, Cd exerted a selection, leading to the drastic decrease of the REF allele in the Cd-exposed population. To get information about mortalities and their temporal dynamic, refer to Pierron et al. (2023 [21]). We have to note that, whatever the condition (Cd or control), no difference in genotype frequencies was observed between females and males (or with larvae, Fig. S5), highlighting the fact that the genotype of individuals did not influence the phenotypic sex of animals. We have also to note that a particular effort was carried out to limit as much as possible potential genetic/family biases. At each generation, we used 20 different mating pairs for each condition. Starting from the F1 generation, spawning pairs were not random to avoid inbreeding (Figure 1). Finally, individuals from different parents were sampled and analysed at each generation.

(a) Changes in the percentage of homozygous alt/alt fish (control, C, blue or cadmium, Cd, red) across generations (F0-F3). Data from adults (males and females, 124 dpf) and larvae (5 dpf) were combined to increase sample size (for each condition and generation, *n* = 18). Different letters denote significant differences (*P* < 0.05, Marascuilo procedure). (b) Schematic representation of the two alleles of the *zbtb38* gene. The genetic variations that we analysed are reported in red. SNP position in NC_007129.7/GRCz11 for ex3: 41212746 and ex4: 41199436, 41199446, 41199619, 41199658. DMR, differentially methylated region identified by MeDip-Seq. Light grey boxes correspond to non-coding regions, dark grey boxes to coding regions, and introns are schematized by a green line.

Since some SNPs were observed in the exon 3 (ex3, non-coding exon) and exon 4 (ex4, coding exon) when comparing the strain TU (GRCz11) and the wild-derived strain NA, we also genotyped fish for these two exons (Figure 2b). We must add that, in the particular case of the ex4, these SNPs were non-synonymous. For the majority of fish (more than 95%), concordant results were obtained. When fish presented the alt sequence in the in2, they also presented the bases referenced in the NA strain. In other words, genetic variations in the in2 were not isolated, but rather two alleles were present (schematized in Figure 2b). This result also underlines that SNPs, even non-synonymous SNPs, were not associated with the phenotypic sex of animals.

#### The exon 3 of zbtb38 encodes for a natural antisense RNA

In order to investigate the potential impacts of the Cd-induced genetic and epigenetic changes on the activity of *zbtb38*, we then analysed transcription levels of the ex2, 3, and 4 of *zbtb38* in zebrafish gonads.

**Figure 3.**
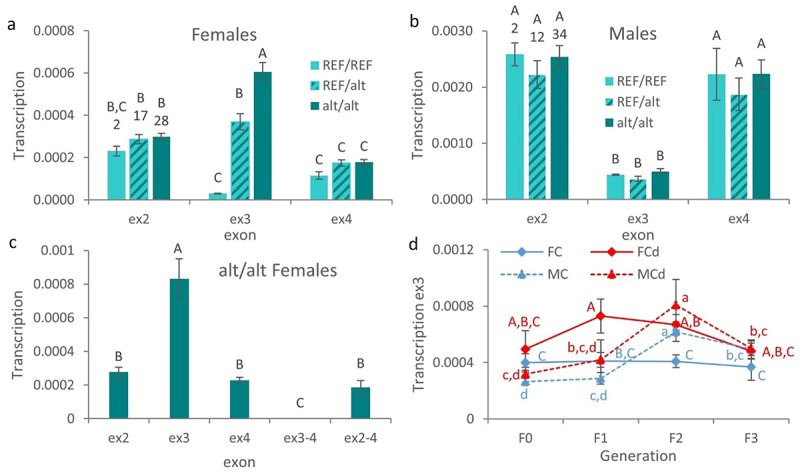
The exon 3 of *zbtb38* is transcribed independently of the other exons.

The only significant effect was observed in the transcription level of the ex3 in females. The transcription level of the ex3 was significantly higher in homozygous alt/alt females in comparison to heterozygous or homozygous REF/REF females (Figure 3a). Moreover, the transcription level of ex3 was significantly higher in heterozygous REF/alt females in comparison to homozygous REF/REF females. Thus, the presence of the alt allele was associated with an up-regulation of the ex3 in female gonads. We must note that such an effect could be linked both to genetic and epigenetic effects as the upstream sequence (in2) showed both genetic and epigenetic (as highlighted by MeDip-Seq) variations between the two alleles (Figure 2b). In this view, we must note that the methylation of *zbtb38* was not found to be affected by Cd in males by MeDip-Seq. In parallel, the transcription level of ex3 in males was not affected by the genotype (Figure 3b), reinforcing the link between epigenetic changes in the in2 and variations in the transcription level of ex3. This obviously requires further investigations and *in vitro* validation.

(a,b) Mean (± SE, *n* = 2–34) transcription levels (a.u.) of the different exons (ex2,3,4) of the *zbtb38* gene in gonads of female (a) and male (b) zebrafish (124 dpf) according to their genotype. Means designated by different letters are significantly different (LSD test, *P* < 0.05), and number of biological replicates of each genotype is indicated upon bars. Fish from the two conditions (C or Cd) were pooled to increase the number of biological replicates. (c) Mean (± SE, *n* = 6, 3 C and 3 Cd female fish) transcription levels of the different targeted regions of the *zbtb38* gene in alt/alt female gonads. Means designated by different letters are significantly different (LSD test, *P* < 0.05). (d) Changes in the transcription level of the exon 3 of *zbtb38* (mean ± SE, *n* = 6) in gonads of female (F, continuous line) and male (M, dashed line) zebrafish from each generation (F0 to F3) and from the two experimental conditions: C, control (blue); Cd, cadmium (red). For each sex, means designated by different letters are significantly different (LSD test, *P* < 0.05).

Furthermore, the transcription level of the ex3 appeared somewhat different from the other exons, suggesting that the ex3 is transcribed independently from the other exons. To test this hypothesis, we then measured the transcription level of different sections of the *zbtb38* gene in homozygous alt/alt females, using primers overlapping different exons (Figure 3c). The obtained results supported our hypothesis. First, no significant difference was observed among the transcription levels of the ex2, ex4, and ex2–4. In contrast, the transcription level of ex3 was significantly higher than that of the other sections. Finally, the transcription level of the ex3–4 section was significantly lower than the other analysed sections and was almost undetectable. In order to get more insight into the ex3 transcript, we used a 3’Race approach using a forward or reverse primer specific to the ex3 with an oligo-dT to identify the end of the transcript. Surprisingly, no amplification was obtained with the forward primer. But, conversely, an amplicon of ~250 bp (which presented 100% homology with the ex3 of *zbtb38*) was obtained with the reverse primer. This result supports the fact that the ex3 is transcribed from the complementary DNA strand (i.e., in the opposite direction of the cognate sense transcript) and suggests that the ex3 transcript is a natural antisense transcript (NAT). Data from the zebrafish reference genome (GRCz11) support the fact that this ex3-NAT is a non-coding transcript (Figure 2b). In zebrafish, three mRNA variants are known for *zbtb38*. The sequence of the ex3 is only detected in the transcript variant X3, where the sequence of ex3 represents the non-coding 5’UTR region of the mRNA.

We then investigated the transcription level of the ex3-NAT across the successive generations (Figure 3d). Its transcription level was significantly higher in Cd females in comparison to control females from the F1 and F2 generations. This can be explained, as mentioned above, by the epigenotype of animals. Indeed, starting from the F1 generation, Cd females were 100% homozygous alt/alt (reaching only 40% at the F0 generation), while control females were mostly heterozygous (the proportion of REF/alt control females from the F1-F3 generation reaching 50%, REF/REF 11.1% and alt/alt 38.9%). We also noticed a significant increase in the transcription level of ex3 in male gonads in both conditions (Cd or control) at the F2 generation. As previously described in Pierron et al. (2021) [10], the only factor that was not similar among generations was population density (i.e., the number of fish per litre). Fish density was two-fold higher at the F2 generation (Tukey’s HSD test, *P* < 0.05) in comparison to other generations. Thus, it appears very likely that population density is responsible for the up-regulation of ex3-NAT transcripts in males at the F2 generation, in addition of being a factor already known to affect the sex ratio in zebrafish [10,29].

### Sex-specific DNA methylation and RNA transcription patterns of zbtb38

**Figure 4.**
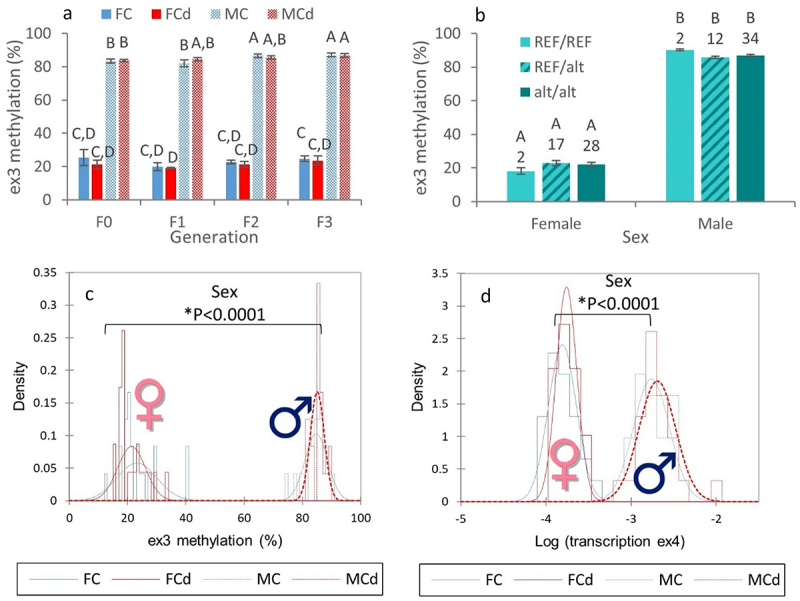
Sex-specific patterns of DNA methylation and RNA transcription.

Since genetic variations within the in2 prevented the analysis of its DNA methylation status by targeted BS-Seq, we then analysed the methylation level of the downstream exon, i.e., the ex3, in adult gonads. Despite the fact that no effect of Cd or genotype was observed (Figure 4a,b), the methylation level of ex3 was significantly influenced by the phenotypic sex. Indeed, a highly significant difference was observed between males and females (Figure 4c). The methylation level of ex3 was significantly higher in males in comparison to females, reaching, respectively, 84.9 ± 0.5% (mean ± SE, *n* = 48) in males and 22.3 ± 0.9% (mean ± SE, *n* = 47) in females. In parallel, transcription levels of the ex2 and ex4 were significantly higher in males in comparison to females (Figure 4d) and were, respectively, 8.5- and 12.3-fold more transcribed in males in comparison to females. These results support the hypothesis that *zbtb38* plays a role in sex determination and/or differentiation and/or maintenance as previously reported for the catfish [22,23]. Interestingly, the higher methylation levels of ex3 in males in comparison to females were associated with higher transcription levels of ex2 and ex4 in males. Whereas up-regulation from methylated genes is not common, there is increasing evidence that DNA methylation does not always act as a repressive mechanism and hypermethylation of promoters and enhancers can be associated with high transcription levels, notably in gonads [19,30,31], by affecting the binding of transcription factors to DNA [32]. We previously reported that the higher methylation levels of the promoter of *foxl2a* in gonads of female zebrafish in comparison to males were associated with higher transcription levels of *foxl2a* in females [10]. Further studies are needed to get further insights into the underlying mechanisms, notably in the promoter region of *zbtb38*. We have also to note that all zebrafish initially develop as females. Then, immature oocytes are either maintained or undergo apoptosis, leading to testis development during the sex differentiation stage that occurs from 21 to 60 dpf [6]. Further studies at different time points are thus needed to ascertain the specific role of *zbtb38*. It is worth noting that if *zbtb38* is regulated, at least in part, by DNA methylation, *zbtb38* itself encodes for a transcription factor that is sensitive to the methylation state of its target, binding methylated promoters in a sequence-specific context. It was recently shown in mammals that ZBTB38 is unique as it has two independent sets of zinc fingers which recognize two different methylated consensus sequences [24]. Even if studies in fish are lacking, genes regulated by *zbtb38* in mammals are known to be involved in cell proliferation, growth, and differentiation [24]. As the methylation state of *zbtb38* is sex-specific and as *zbtb38* regulates the transcription levels of other genes via the methylation status of their promoters, *zbtb38* could play a non-negligible role in sex differentiation by participating in the orchestration of the sex-specific transcriptome. This obviously requires further study, notably by investigating the relationship between *zbtb38* and the other genes already known to be involved in sexual differentiation.

(a,b) Changes in the methylation level of the exon 3 of *zbtb38* in gonads (a) of female (F, full bars) and male (M, hatched bars) zebrafish (124 dpf) from each generation (F0 to F3) and from the two experimental conditions: C, control (blue); Cd, cadmium (red). Means (± SE, *n* = 6) designated by different letters are significantly different (Conover–Iman, *P* < 0.05) or (b) according to the sex and genotype of fish. Fish from the two conditions (C or Cd) were pooled to increase the number of biological replicates. Means (± SE, n is indicated upon bars) designated by different letters are significantly different (LSD, *P* < 0.05). (c,d) Distribution of (c) ex3 methylation levels and (d) ex4 transcription levels (expressed as Log) in gonads of adult fish according to the sex (Female, F; Male, M) and condition (Control, C; Cadmium, Cd). Data from the two conditions (*n* = 47–48 per sex) were combined to investigate the effect of sex, and results of the consecutive Student’s T tests are indicated.

### zbtb38-NAT is associated with changes in offspring sex ratio

**Figure 5.**
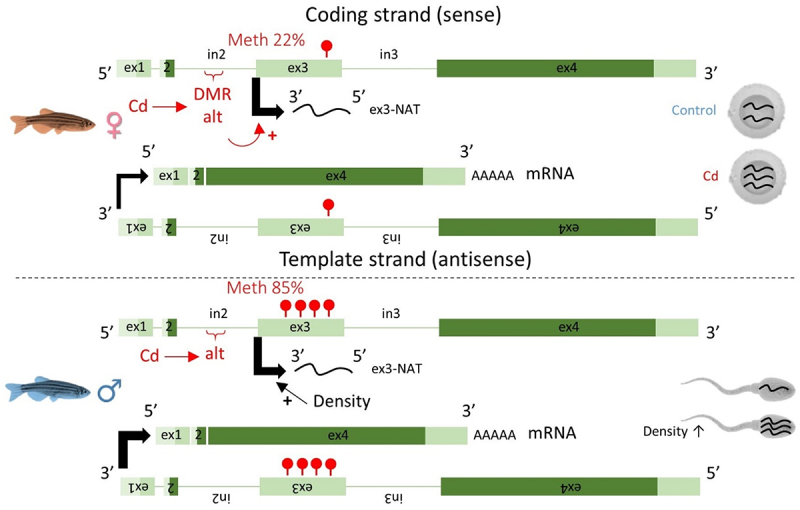
A complex dialog between the environment, the genome and DNA methylation led to variations in the transcription level of the ex3-NAT of *zbtb38.*

Our results highlighted sex-specific patterns of both DNA methylation and transcription of *zbtb38* in sexually mature individuals (124 dpf), supporting a role of this gene in sex differentiation and/or maintenance. We also discovered that the ex3 is transcribed from the complementary DNA strand. In contrast to the other exons, the transcription level of this ex3-NAT was not sex-specific (Student’s Test, *P* = 0.54, *n* = 47–48 per sex) but appeared to be influenced by environmental cues: by Cd *via* epigenotype variations in females and by density in males, leading to variations in the NAT content of gametes across the successive generations (Figure 3d). Results are summarized in Figure 5.

**Figure 6.**
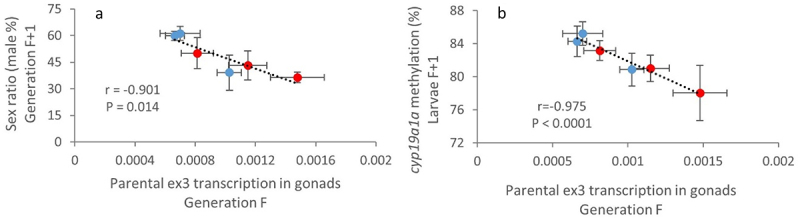
Parental expression of ex3-NAT influences offspring sex ratio.

Next-generation sequencing has enabled the discovery of a vast amount of non-coding RNA (ncRNA) from yeast to humans. Despite NATs are one of the most poorly understood ncRNA species, recent evidences support an essential role of NATs by their involvement in gene regulation [33,34]. Increasing evidence also suggests that ncRNAs are sensitive to environmental factors and contribute to the inheritance of environmentally induced phenotypes [35–37]. More specifically, paternal reproductive influence on offspring *via* the ncRNAs content of sperm has gained attention in recent years [38,39]. In our case, considering the potential involvement of *zbtb38* in sex differentiation and the transgenerational feminization of the Cd-exposed population, we tested for a potential relationship between the transcription level of the ex3-NAT in adult gonads (F generation) and the sex ratio of their offspring (F + 1 generation). We observed a significant relationship between the paternal transcription level of ex3-NAT and the sex ratio of their offspring (Pearson correlation coefficient *r* = −0.884, *P* = 0.019). Despite the fact that the relationship was not significant for females (*r* = −0.609, *P* = 0.2), a much more significant relationship (*r* = −0.901, *P* = 0.014; Figure 6a) was observed when we cumulated the mean transcription levels of ex3-NAT in males (paternal) and females (maternal). Since previous works reported a key role of the methylation status of the *cyp19a1a* gene in sex differentiation in fish [10,15], we also tested for a potential relationship between the parental expression of ex3-NAT and the methylation level of *cyp19a1a* in their offspring (assessed in whole larvae, see Pierron et al., 2021 [10]). A highly significant correlation (*r* = −0.975, *P* = 0.0009; Figure 6b) was observed. This result reinforces the fact that the parentally transmitted ex3-NAT transcript can influence the sex of the future embryos.

(a,b) Relationship between the mean transcription level (± SE, n = 12) of the exon 3 of *zbtb38* in gonads of parents (mean transcription levels from females and males (124 dpf) were added, n = 6 per sex) from each generation (F) and (a) the sex ratio at the next generation (F + 1) (males %, mean ± SE, n = 4 aquaria per condition, number of fish per generation and condition ranged from 303 to 663) or (b) the mean methylation level of *cyp19a1a* in whole larvae (5 dpf, n = 6) of the next generation (F + 1). The Pearson correlation coefficient (r) and the associated *P* value are reported.

Although this field of inquiry is still in its infancy, it is known that NATs regulate gene expression by several mechanisms, not only by interfering with their cognate sense transcript but also by modifying epigenetic marks (DNA methylation and histones) and by playing an essential role in gene imprinting [33,34]. Interestingly, the most prominent examples in mammals rely on sex genes/chromosomes. In humans, suppression of Tsix (an antisense to Xist) leads to ectopic expression of Xist and concomitantly to X chromosome inactivation [33]. Imprinted genes are expressed preferentially from the paternally or maternally inherited allele. In mice, the *igfr2* gene is paternally imprinted (the maternal allele is transcribed) and its imprinting is regulated by a DMR (located in the intron 2) which functions as a promoter for a NAT called *airn* [40], a mechanism that could present some similarities with what we observed for *zbtb38* (Figure 5). However, we must note that if gene imprinting has been found in mammals, there is no evidence so far for imprinting in non-mammalian vertebrates.

## Conclusion

The transcription level of the *zbtb38* mRNA in fish gonads was found to be sex-specific. Moreover, while *zbtb38* encodes for a transcription factor that is sensitive to the methylation state of its target, the methylation level of *zbtbt38* was itself very different between males and females, highlighting complex regulatory mechanisms. Further complicating the matter, we also discovered that the ex3 of *zbtb38* encodes for a NAT, coupling at least two epigenetic mechanisms, i.e., DNA methylation and ncRNA. We also observed that (i) the transcription of this NAT is influenced by environmental cues and (ii) increasing transcription levels of this NAT in gametes of parents is associated with a high proportion of females in their offspring. Our results thus support a non-negligible role of *zbtb38* in sex differentiation and suggest that the environment experienced by parents can affect the sex ratio of their offspring via a complex epigenetic dialog.

## Supplementary Material

Supplemental Material

## Data Availability

Raw sequencing data were deposited in the European Nucleotide Archive with accession number PRJEB52137. All other data are included in the article and/or supporting information.
