# Supplementary material for: Sex-specific DNA methylation and transcription of zbtb38 and effects of gene–environment interactions on its natural antisense transcript in zebrafish
Source: Epigenetics. 2023 Oct 2;18(1):2260963. doi: 10.1080/15592294.2023.2260963 (PMC10547075; doi:10.1080/15592294.2023.2260963)
Supplement: Supplemental Material [file KEPI_A_2260963_SM1203.docx]

**Supplementary Material for:**

**Title: Sex-specific DNA methylation and transcription of zbtb38 and effects of gene-environment interactions on its natural antisense transcript in zebrafish**

**List of authors:**

Fabien Pierron^*^, Flore Daramy, Débora Heroin, Guillemine Daffe, Aurélien Barré, Olivier Bouchez and Macha Nikolski

***Corresponding author:**

Fabien Pierron

UMR 5805 EPOC - OASU

Station Marine d'Arcachon

Université de Bordeaux

Place du Docteur Bertrand Peyneau

33120 ARCACHON

France

Phone : +335 56 22 39 33

[fabien.pierron@u-bordeaux.fr](mailto:fabien.pierron@u-bordeaux.fr)

**This PDF file includes:**

7 pages

Table S1 (p. S2)

Figures S1 to S5 (p. S3-S7)

**Table S1:** Primers used for (i) sequencing by Sanger (PCR_Seq), (ii) genotyping by qPCR (qPCR_genotyping) or by PCR and capillary electrophoresis (PCR_genotyping), (iii) for DNA methylation analyses by bisulfite sequencing (PCR_BSseq), (iv) for transcription analyses by RT-qPCR (qPCR_transcript) and (v) 3’RACE. F forward; R reverse; S sequencing; sequences in bold are biotinylated in 5’.

| **Region amplified** | **Analyze** | **Sequence position on GRCz11** | **Primers** |
| --- | --- | --- | --- |
| ***zbtb38***  **Intron3** | PCR_BSseq | NC_007129.7  41216235-41216400 | F: AGTTTTTTTGTTAAGGGGAAAATGT  R: **CCTCCACTAAAAATAACAAACTTTCACA**  S: GTTAAGGGGAAAATGTT |
| ***zbtb38***  **Intron3** | PCR_Seq | NC_007129.7  41215998-41216801 | F: TTGACACTTTGATTAACTTGTAGCC  R: CATTTGCATACAGAGCAGTGG |
| ***zbtb38***  **Intron3_REF** | qPCR_genotyping | NC_007129.7  41216038-41216343 | F: GCCCCTAGAAGGTCTTGGAC  R: AGACCAGTACCGGTGGATCA |
| ***zbtb38***  **Intron3_alt** | qPCR_genotyping | / | F: CTTGCGGCGCGCTAAC  R: GAACCCACCAGATCTGTCCAAGA |
| ***cat***  **Exon8** | qPCR_genotyping | NC_007136.7  7498200-7498299 | F: GTTCCCTCTGATTCCTGTGG  R: TGGCATGTTACTGGGATCAA |
| ***zbtb38***  **Exon4_REF** | PCR_genotyping | NC_007136.7  41199423-41199667 | F:AGGTGATAAAGGCATAGTGTAGCC  R:GACCAGAGGGCATAGGTCAA |
| ***zbtb38***  **Exon4_alt** | PCR_genotyping | / | F:AGGTGATAAAGGCCTAGTGTAGCA  R:AATCCAAGTTTGCGGTTGAG |
| ***zbtb38***  **Exon3** | PCR_BSseq | NC_007136.7  41212711-41212837 | F:TGGGTTTATTTTTAATATGGTTGTTGAT  R:**AATATAAAACAACTCCTCCCTATTT**  S:ATATGGTTGTTGATTGATGT |
| ***zbtb38***  **Exon2** | qPCR_transcript | NC_007136.7  41218962-41219034 | F: GCACAGTGGAGTCGTTCCTC  R: CGAGATCGTCTTTCCTCTGG |
| ***zbtb38***  **Exon3** | qPCR_transcript  3’RACE | NC_007136.7  41212006-41212087 | F: AACCGGAGGTTGAGTTGTTG  R: CGAGGAAAATGGATCAGCAT |
| ***zbtb38***  **Exon4** | qPCR_transcript  3’RACE | NC_007136.7  41199029-41199096 | F: CAGCCTAAGCATGGAAAAGC  R: TGGTGGCAAGGTAAAGGAAC |
| ***zbtb38***  **Exon3-4** | qPCR_transcript  3’RACE | NC_007136.7  41211519-41202230 | F: GAGCGATGTTTTTCGCTCTC  R: AGAGTGCTTGGGTTCACCAC |
| ***zbtb38***  **Exon2-4** | qPCR_transcript | NC_007136.7  41218966-41202230 | F: GGGACCAGAGGAAAGACGAT  R: AGAGTGCTTGGGTTCACCAC |
|  | 3’RACE |  | Anchored oligo-dT:  AAGCAGTGGTATCAACGCAGAGTTTTTTTTTTTTTTTTTTTTVN  R:AAGCAGTGGTATCAACGCAGAGT |

**Figure S1:** Pyrograms obtained for the exon 3 of the *zbtb38* gene using bisulfite-treated DNA.

The sequence analyzed after bisulfite treatment was as follows:

AT/AAGGGATGYGGTYGTTTTGTAGAGYGTTGGAGAGGGAGGAGATGGGAGGG

Where T/A corresponds to a SNP (T in GRCz11 (REF sequence) and A in the strain NA (alt sequence))

Y = C or T = methylation site


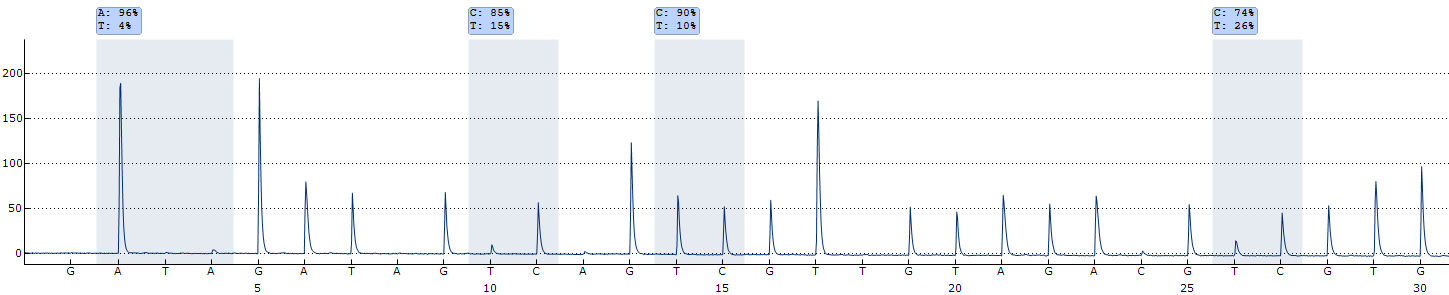

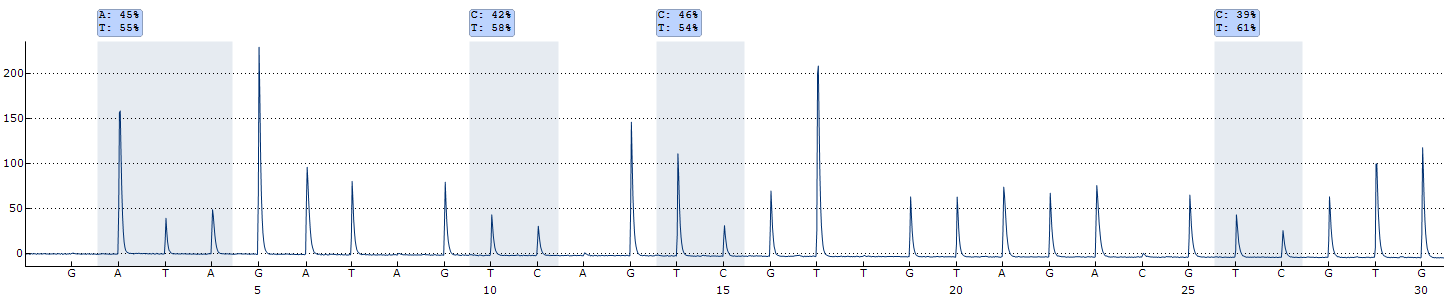

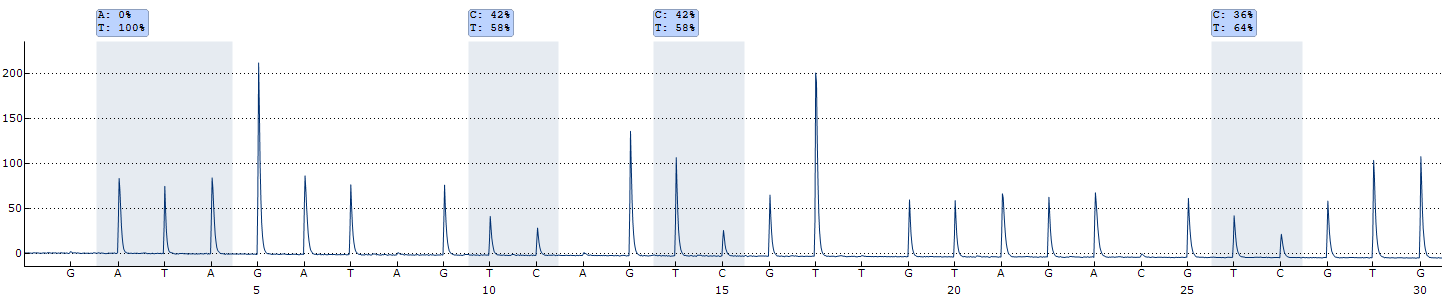


SNP

REF/REF

REF/alt

alt/alt

CpG1

CpG2

CpG3

**Figure S2:** Genotyping by qPCR of the intron 3 of the *zbtb38* gene

In order to determine the genotype of individuals for the intron 3 of the *zbtb38* gene, we designed specific primers for the REF and alt sequence. We also amplified a sequence located in an exon of the catalase gene as an internal reference.

(A) Alignment of the REF and alt sequences and location of the primer pairs (underlined sequences) used to specifically amplify the REF (blue) or the alt (red) sequence (see Table S1 for primers). (B) Examples of qPCR results obtained for individuals with different genotypes.


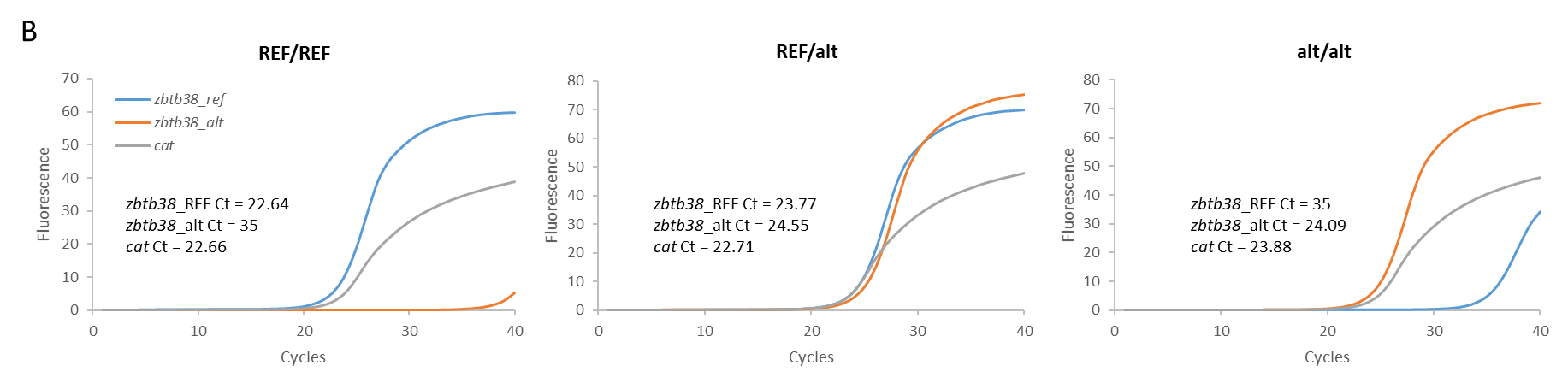


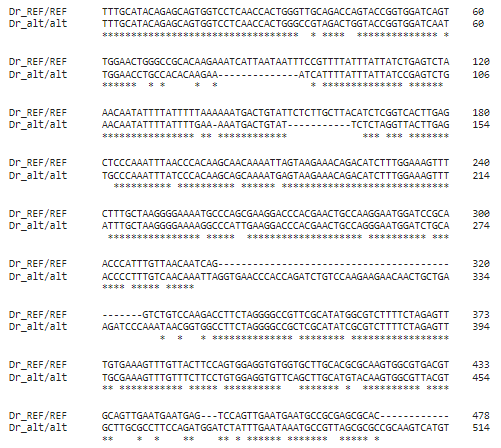


A

**Figure S3:** Genotyping of the exon 4 of the *zbtb38* gene by PCR and capillary electrophoresis

(A) Four SNPs (green) were identified in the exon 4. Primers (underlined sequences) were designed to amplify specifically the REF (blue) or the alt (red) sequence. PCR products were migrated and visualized by means of a QIAxcel instrument (Qiagen). (B) Examples of results obtained for individuals with different genotypes.

**A**

>REF_GRCz11_|NC_007129.7|:41199423-41199667

**AGGTGATAAAGGCATAGTGTAGCC**TCTCATGTGTTTCATTTTTATTCGTTTGCTTTTCAGCTTCTTACTCCTCTTTTTAGGCTTATATGTGTAATAGGGACGCCATAGTTCATCAGTTTCGGGATCGTTTGCGTCATCATATGTTTCTTTTTCTCTAACTGAGGTTGTGACTTCTGCCCTCAACCGCAAACTTGGA**C**TTCCAATACTCTCCTCTGGACGGTTACT**TTGACCTATGCCCTCTGGTC**

>ALT_NA_LR812586.1_40855373-40855617

**AGGTGATAAAGGCCTAGTGTAGCA**TCTCATGTGTTTCATTTTTATTCGTTTGCTTTTCAGCTTCTTACTCCTCTTTTTAGGCTTATATGTGTAATAGGGACGCCATAGTTCATCAGTTTCGGGATCGTTTGCGTCATCATATGTTTCTTTTTCTCTAACTGAGGTTGTGACTTCTGCC**CTCAACCGCAAACTTGGATT**TCCAATACTCTCCTCTGGACGGTTACTTTGACCTATG**A**CCTCTGGTC

**
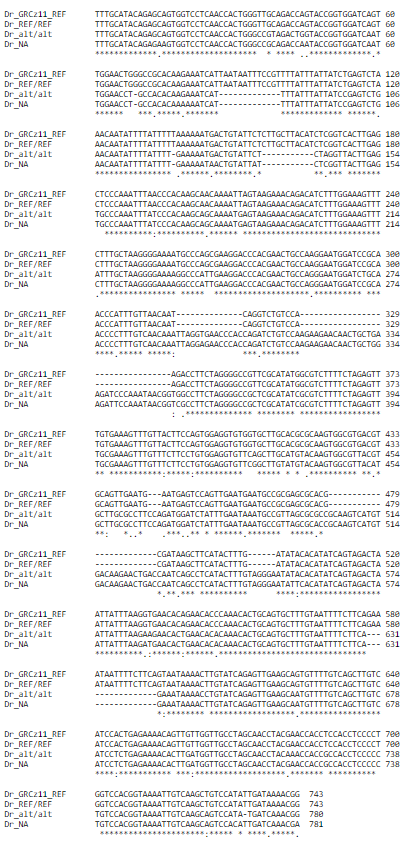
Figure S4:** Alignment of the sequences obtained for the intron 2 using native (untreated DNA) as template. Dr_GRCz11_REF refers to the reference sequence available in the zebrafish reference genome (GRCz11), Dr_REF/REF to the sequence obtained after sequencing of a homozygous REF/REF fish, Dr_alt/alt to the sequence obtained after sequencing of a homozygous alt/alt fish and Dr_Na to the sequence obtained from the genome of the wild-derived strain Nadia. Alignment was carried out with Clustal-omega.

REF primers

alt primers

Sample 449 = REF/REF

Sample 457 = REF/REF

Sample 465 = alt/alt

Sample 174 = REF/alt

Sample 433 = alt/alt

Sample 441 = alt/alt

B

**Figure S5:** Change in genotype frequencies (intron 2) according to the life stage (larvae L), sex (Male, M and Female, F) and condition (Control, C or cadmium, Cd). Data from the 4 generations were combined to enlarge sample size. Different letters denote significant differences, homogeneity chi-square test (P<0.05).
